# Eating Experiences of People with Disabilities: A Qualitative Study in Spain

**DOI:** 10.3390/healthcare8040512

**Published:** 2020-11-24

**Authors:** Carmen Cipriano-Crespo, Marta Rodríguez-Hernández, Pablo Cantero-Garlito, Lorenzo Mariano-Juárez

**Affiliations:** 1 Faculty of Health Sciences, University of Castilla La Mancha, 45600 Talavera de la Reina, Spain; MariaCarmen.Cipriano@uclm.es (C.C.-C.); Pablo.Cantero@uclm.es (P.C.-G.); 2 Faculty of Nursing and Occupational Therapy, University of Extremadura, 10003 Cáceres, Spain; lorenmariano@unex.es

**Keywords:** disability, occupational therapy, food, qualitative research

## Abstract

Background: Disability causes changes in the eating process, which is central to the definition of each individual’s social and psychological spaces. Methods: This is a qualitative study based on grounded theory. Interviews were carried out in clinical hospital settings and headquarters of several disability organisations. The study included 27 individuals, aged between 18–75 years. All participants had a disability that caused a functional deficiency in the occupational aspects of the eating process. Results: The respondents’ narratives were analysed through observations made in different contexts, allowing us to describe and understand the significance attributed by the participants to their reality and experiences. Three key themes emerged from the analysis: (1) waning bodies (assumption of a diminished corporality); (2) redefinition of food-related social spaces; and (3) perceived burdensomeness, shame, and loneliness. Conclusions: Assisted feeding tends to prioritise the nutritional component of food ingestion. However, cultural, social, and contextual factors have a critical impact on an individual’s well-being and quality of life. This study stresses the importance of re-addressing intervention models affecting differently-abled people and incorporating approaches that take into account the contextual aspects of occupational therapy.

## 1. Introduction

Eating is an essential component of human occupational activities [1]. Food-related topics are frequently mentioned in interventions with groups of people with autism or developmental disorders, either regarding their positive psychosocial effects [2,3,4] or as a way of promoting healthy habits. Occupational therapists regularly take part in community programs focused on nutritional practices [5]. Studies of people with disabilities have sometimes addressed problems like food insecurity in households or difficulties in accessing food [6,7].

Most approaches to food and eating issues among people with disabilities, however, have focused on the nutritional elements of the process and the patients’ biological (medical) needs [8]. Occupational therapy literature tends to focus on occupational performance aspects―physiological processes, such as chewing or swallowing, as well as issues related to cognitive, motor, or postural capabilities, or problems like dysphagia [9,10]. Ochoa et al. [11] analysed the perception of food among people with Parkinson’s disease, examining the emotions they experienced while eating in public places or during social events. Lance [12] studied several people with disabilities who depended on others for assisted eating, describing withdrawal patterns in certain social circumstances. Morley and Neufeldt [13] described the emotions, social roles and personal journeys experienced by several patients, including some with cancer. Liu in 2019 [14] suggested a pattern of reduced social engagement among older adults with disabilities. Although there are more examples, the attention received by these issues is comparatively low.

Besides nourishing the body, eating activities create a space for social interaction, and are closely entwined with definitions of the self and self in society. As a central component of human activity, their social, cultural, and individual implications are as relevant as the nutritional ones, and it is important that this point gets the recognition that it deserves. This is the approach advocated by those who consider that qualitative approaches based on patients’ personal experiences are first-rate evidence, as our study does―what has been described as narrative-based medicine [15,16,17].

This study aims to describe people with disabilities’ experiences in their adapting to new food-related occupational spaces, and the importance of the social and individually experienced aspects of the feeding and eating process. It also underscores the importance of integrating these viewpoints into the research and professional practice of occupational therapy.

## 2. Materials and Methods

This is a qualitative study based on grounded theory [18]. Data collection and analysis followed an inductive approach [19]. Categories were established following the analysis of raw empirical material, using the constant comparison method within the theoretical framework defined. This allowed for a better analytical understanding of the experiences (illnesses) of a group of individuals with disabilities that presented feeding difficulties [20].

### 2.1. Participants

The study included 27 individuals (21 males, 6 females), aged between 18 and 75 years. All participants had a disability that caused a functional deficiency in the occupational aspects of the eating process (stroke, spinal cord injury, cancer, multiple sclerosis, Duchenne muscular dystrophy, acquired brain damage, amyotrophic lateral sclerosis, or Niemann–Pick disease) (Table 1). Some had difficulty chewing or swallowing food, and some were incapable of using cutlery to feed themselves and needed help. Those with cognitive impairments, those who did not give consent, and those who refused to be audio-recorded were excluded from the study.

### 2.2. Data Collection

Data collection was based on in-depth interviews, guided by a series of semi-structured questions (Table 2), which allowed the exploration of new themes emerging during the course of the interviews [21]. All interviews were audio-recorded, and notes were taken in a field journal for their duration [22]. Interviews were carried out between January and December 2019 in different settings (speech therapy clinics, hospital rehabilitation units, and headquarters of disability organisations). Efforts were made to facilitate comfort and privacy during the interviews, helping to provide an environment that fostered a candid conversation between participants and researchers. All interviews were carried out by the same researcher and lasted between 60–120 min.

Each audio recording was assigned a code name, in order to preserve the participants’ privacy and confidentiality, while maintaining the emotional character of the interviews and the link between each individual and their biography. In order to guarantee privacy, all personal data that could reveal the participants’ identity have been eliminated from this article. The original audio recordings were destroyed once they had been transcribed.

### 2.3. Data Analysis

Interviews were transcribed and analysed in tabular datasheets. Empirical material was analysed and interpreted in light of existing social theory and the theoretical occupational models considered. Data analysis was undertaken by two experienced qualitative researchers, who worked independently. Once their analysis was complete, they shared and discussed their results. The participants’ narratives were identified and analysed through systematic content coding: (1) preliminary units of analysis were established after a first reading of the transcripts; (2) using the constant comparison method, open-coded data were grouped into concepts, and categories were generated accordingly. Initially, 22 categories were defined, which were later grouped into ten categories. Once data had been broken down, relationships between categories and subcategories were actively and systematically analysed in a process of axial coding (Table 3) [22,23]. Finally, following a process of selective coding, categories were grouped into themes that, in line with the aims of the study, described the participants’ experiences and the meanings attributed to these. 

Different triangulation techniques were applied to guarantee the data quality, validity, and accuracy: (1) across researchers—the interviews were carried out and analysed by different specialists; (2) across methodologies—different techniques (in-depth interviews and field notes) were used for collecting data on the participants; and (3) across the different theoretical approaches on which the inductive method was based.

Throughout the research process, to guarantee the quality and validity of the study, the authors followed the criteria defined in the COREQ (consolidated criteria for reporting qualitative research) checklist for reporting qualitative research, as well as the verification checklist [24].

### 2.4. Ethical Considerations

This study was approved by the Clinical Research Ethical Committee of the Talavera de la Reina Integrated Management Area (CEIm del AGI de Talavera de la Reina in Spain, Hospital Nuestra Señora del Prado, ref: 18/2014). The research was in line with the ethical principles outlined in the Declaration of Helsinki and the Belmont Report.

## 3. Results

The disruption caused in a person’s life by disability affects everyday practices, altering habits and subjective experiences. From an occupational viewpoint, the main therapeutic concerns regarding patients’ eating patterns are recovery and making progress regarding physical (biological) indicators―that is, eating is only considered in terms of occupational performance and nutritional intake. For most of the respondents in this study, our questions about their feelings and experiences were the first time interest had been expressed about those issues. Professional care practice tends to describe the new reality of people with a disability around the idea of loss: it is necessary to work out ways to ensure that patients can still eat and “get used” to their new eating situation. However, this “psychological adaptation” approach fails to understand the importance of those significant spaces for the individual with a disability, or their role in their personal identity construction processes. 

**Figure 1:**
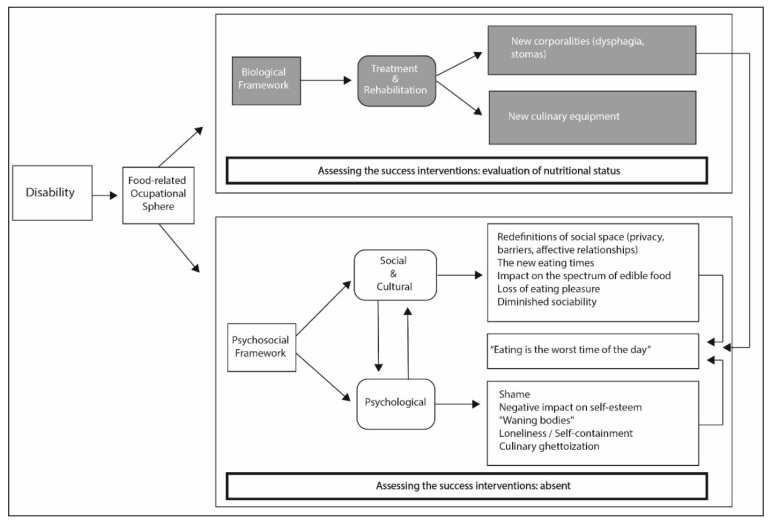
Impact of disability on the occupational sphere. Therapeutic concerns are highlighted in shadowed areas.

The analysis of our empirical materials revealed three key, recurrent themes, which emphasise the importance of occupational activities for people with disabilities: their increased sense of “diminished corporality”, the processes of redefinition of the social spaces associated with food, and the emotions and habits that are built around social, cultural, and psychological interactions (Figure 1).

### 3.1. Waning Bodies: Assumption of a Diminished Corporality

One of the main effects of disability in the occupational eating sphere is related to bodily changes, caused either by illness or medical interventions. The body is different, which causes changes regarding what food can be consumed and how. Also, beyond this evident physiological impact, there are also psychological problems and fears—for instance, in the case of dysphagia (a frequent issue), the fear of not knowing how to eat or fear of choking. For the respondents in our study, their mouths no longer felt like theirs, which undermined their very notion of “self”. Their mouths, throats and, by extension, their bodies, could no longer be trusted. Leoncio, who underwent a laryngectomy due to laryngeal cancer, was forced to break up filini (an extremely thin variety of pasta) before cooking it:

*I had to break them, I used to do it before cooking them―it was almost an engineering procedure since they were already the thinnest kind of noodles, but I had to do it to avoid feeling pain.*

Having to devise new ways of eating created a feeling of nostalgia about the past, thus cementing the narrative of loss—of the pleasure of eating, and of the meaning of eating. This caused an increased perception of their bodies as defective, waning, or diminished.

We live in times of hedonistic attitudes toward food [25,26]. Our choices of food are based on a desire for pleasure and satisfaction, and this is an issue that dominated discourse and practice among our respondents. For this reason, the loss of the ability to eat and taste food was a real tragedy, to the extent that some of our respondents described attempts to recover it, despite the risk of death that it could pose. In their own words, they needed to feel they were eating “properly”. Such is the social and symbolic importance of food.

*Sometimes, at home, I go to the fridge and grab a bit of ham because I cannot stand it anymore to see food and not being able to swallow it [...] When I do this I feel I have eaten properly, I feel satisfied because I have eaten, but it is a feeling compounded with a fear of choking.*

(Jesús, 70 years old. Laryngeal cancer)

Physical changes are usually involuntary, and they produce severe alterations to eating routines. Most of our respondents described their new situation as an “artificial way of eating.” Herrera [27] has described this process as one of agency reversal, from a body that eats to a body that needs to be fed. Javier, who had had a laryngectomy due to laryngeal cancer, described the process as “food being poured into him” (the metaphor itself is revealing), and how this did not make him feel satiated. Eating because of medical prescriptions is something else altogether:

*Although I do not feel hungry, they feed me because I have to be fed, and that’s that. It does not feel like I am eating [...] The way I eat nowadays fills me up, sometimes it stops me feeling hungry, but I do not feel that I am eating since I cannot taste the food, I do not chew or swallow it, it is completely artificial.*

(Javier, 65 years old. Laryngeal cancer)

Sometimes, the impact of the illness is realised as a consequence of anosmia, which can cause problems like loss of appetite and the inability to enjoy food [28]. Olfactory cues play a key role in the experience of food, and they can also trigger olfactory memories and nostalgia. Our interviews revealed how this problem shaped the respondent’s occupational experiences. Mariano explained how, after radiotherapy, food started to taste “like lead [...] it felt like some of the food had crystals in it.” He was unable to taste anything, and he did not enjoy eating. Jesús Javier, who worked with animals, admitted that not being able to assess his own smell caused him anxiety: “I can be smelly without knowing it, my wife sometimes has to tell me to wash because I still smell of cattle―it is humiliating”.

### 3.2. Social Redefinition of Food-Related Spaces

The disruptive effect of disability also affects eating spaces, blurring the line between public and private behavior and transforming the “self” into “self with help”. Research literature has examined in detail the impact of healthcare institutions on the loss of privacy. However, the effect of the loss of self-reliance in the definitions of the self has not received the same degree of attention. For instance, in the eating sphere, new actors appear who decide what needs to be eaten, when, and how:

*[...] the carers lost patience and ended up feeding me themselves. They made me eat everything, and afterward I felt unwell. [...] They told me off all the time―if I ate too slowly, if I did not eat, and eventually they fed me themselves. They made me eat too fast, and the food did not sit well.*

(Benito, 71 years old. Acquired brain damage)

The new configuration of the eating space is defined by the presence of others, who decide on the time frame and the space where feeding must occur, without being altogether aware of the impact of their interference in their patients’ subjectivities. Malterud and Thesen [29] noted in their study the lack of interest or awareness of some health professionals about the sensory aspects of the feeding process, neglecting basic needs while dutifully cramming food into a patient’s mouth. Our respondents suggested that the loss of their privacy negatively affected their self-esteem and, as we mentioned above, made them see their bodies as defective or less valid. 

However, the limitations on the eating sphere are not only caused by medical interventions. Venturiello suggested that “streets, buildings, ramped accesses, sidewalks, squares, lifts, and toilets mediate the social networks” [30] within which the respondents conduct their interactions. Accounts of eating out, away from the familiar home environment, always emphasise the preparations required—plans to anticipate potential problems and the careful choice of destinations. The locations of toilets in bars and restaurants often create barriers for people in wheelchairs, such as María, who suffers from muscular dystrophy. To avoid this problem, she decided to carry a plastic bucket with her in case she needed to use the toilet:

*[...] would leave my bottom right on the edge of the chair, putting the bucket underneath so it would catch the pee. This way I could go to the bars and restaurants that I liked, and I was allowed to do this, and my husband did not have to carry me on his back any more to climb the stairs to the toilet.*

(María, 50 years old. Muscular dystrophy)

Eating spaces are also pared down, transformed by the loss of emotional associations. Culinary spaces, such as the kitchen or the pantry, are, in Spanish tradition, social spaces closely entwined with affective relationships, particularly for women 

However, the filter of disability made them be perceived as cold spaces, like pharmacies or laboratories, full of aid equipment or cold prepared meals, with no associated memories or meaning. Occupational therapy prescribes aids and prepared meals in order to minimise functional difficulties for people with disabilities, and to facilitate maximum independence. From a clinical point of view, they are considered as valuable and necessary tools. From an emic viewpoint, however, the reality is very different―they are part of new culinary equipment that is often seen as a source of grief. Isaac, who has multiple sclerosis, uses a stabilising spoon to control his tremor. However, he avoids using it in public places, where he can be seen, even though he admits that this makes his eating messier, which in turn increases the negative perception of people with his disability. Aid equipment, perceived as cold, unaesthetic, and not normal, can cause disaffection and is the subject of ambivalent feelings: while it allows a more independent living, it can also be seen as a performative component of stigmatisation. 

Similarly, invasive feeding devices, such as a PEG tube placed through the abdominal wall into the stomach or a gastrostomy button, allow a patient to be fed, but the process does not bear any resemblance to what could be considered as “culinary normality”. Instead of considering plate presentation, feeding times now include a variety of plastic cutlery, rubber feeding tubes, and digital devices that speak volumes about the radical changes in their lives. Dining rooms are no longer spaces for emotions and affects, but rather like cold, medicalised hospital rooms.

*Preparing food was rather sad and repetitive, first everything had to be finely blended and it was really hard, and then it had to be added to the mix and the smell was horrible, like medicine, not appealing at all―yet I had to feed it to my son because there was nothing else he could have.*

(Isabel, Samuel’s mother. Samuel, 18 years old, suffers from Niemann–Pick disease)

Finally, the narrowing down of the choice of eating spaces is not only due to the structural problems encountered in public places. At play are also processes of social withdrawal and self-isolation, caused by self-consciousness about their new feeding requirements.

### 3.3. Burden, Shame, and Loneliness

When we eat, we are participating in both a social and a socialising activity [26]. This is particularly true in Mediterranean countries, where the tradition of postprandial conversation is as important as the meal itself. However, this was no longer the case for our respondents. Notions of shame and embarrassment underpin patterns of social withdrawal:

*While I eat I drop a lot of food and once I heard a man saying aloud that it was disgusting, and he turned his back on me so he would not see me. Since that day, I prefer to eat when I am alone.*

(Paloma, 60 years old. Amyotrophic lateral sclerosis)

A reduction in social interaction is frequently described in analyses of the subjective experiences of new feeding requirements [31,32,33]. Our research revealed a process of “culinary ghettoisation” that not only affected the frequencies of eating out, but also narrowed down the physical spaces and the times in which it was possible to eat: in the kitchen or in the dining room, when nobody else could see them.

The processes of self-isolation are dominated by a desire to avoid being perceived and labeled as “childish” for an inability to maintain table manners and for having a particular pace that others do not understand. Many of the respondents in this study admitted feeling like a “burden” for others. Some respondents described giving up on the social, festive aspects of eating—a central theme in this study.

*Because of my issues we do not celebrate anymore, everybody knows that not being able to eat like they do is a source of grief, so to avoid it we do not celebrate with food, which is what we used to do in my family―like almost everybody else does, this is Spain!*

(Javier, 65 years old. Laryngeal cancer)

## 4. Discussion

Occupational models of eating practices [34] take into account, at least theoretically, the multidimensional nature of the process. The process of eating involves more than physical and physiological needs—it must be considered from a holistic perspective that includes aspects of identity, as well as social and cultural components [35,36], transcending the classical categories of self-care, productivity, and leisure that occupational therapy has traditionally prioritised [37]. It is illustrative that studies that address the social and cultural elements of the eating process, rather than focusing on the body and related physiological issues, are almost negligible by comparison [36,38].

The interviews analysed highlight the importance of taking into account sociocultural needs as well as physical ones, in terms of nutritional requirements and calorie intake. Our respondents’ experiences suggest that the social, symbolic, and psychological aspects of the eating process are being neglected under the imperative of needing to “adapt psychologically” to a new reality. Their narratives, however, emphasise how important those needs are. Malmström et al. noted the transformation in the significance attributed to food and the act of eating when a leisure activity was turned into a constant, daily struggle to avoid losing control of a vital activity [32]. The participants in this study described with sorrow their feelings of loss―of their previous eating habits, of the emotions and pleasures that food evoked, and of their usual eating spaces. This had a severe impact on issues like personal identity construction and self-esteem, with strong feelings of shame and being a burden causing processes of social withdrawal and self-isolation. Previous studies, such as those of Winkler and Ottoson, have pointed out that the experiences of interaction with others can help define personal identities, and thus for people with feeding difficulties the spaces for socialisation are reduced [39,40,41]. 

At the same time, it is essential for clinical narratives to admit that people with a disability affecting food ingestion of food still feel a need to “eat properly”, even if it poses a risk to their lives. Illnesses do not only affect the body and how food is consumed, but the whole of an individual’s life [41].

The results of this study have an enormous relevance for clinical practice. On the whole, intervention models regarding food and feeding practices for people with disabilities, or those who are experiencing eating difficulties due to medical treatment, tend to focus on the nutritional aspects of the process. However, the narratives analysed in this study emphasise the importance attributed to social and symbolic elements. At the same time, it is important to examine in detail the consequences of the new eating situation on contextual relations and the definitions of the self, since these issues have a tremendous impact on the patients’ health. It is necessary to take into account culinary loneliness, which can be, as has been suggested, detrimental to mental health [28], as well as the new occupational needs created by the narrowing down of physical spaces and the increased sense of damaged corporality.

The results of this study suggest that new intervention models, based on the valorisation of how people with disabilities experience food, are necessary. The importance attributed by the participants to these subjective aspects requires a re-examination of current clinical practices [17], and the inclusion of patients’ experiences in the intervention protocols followed by occupational therapists. 

Despite its limitations, this study has suggested a possible approach to explore differently-abled individuals’ personal experiences. However, it is important to consider that participants in the study had very different kinds and levels of disability, caused by diverse pathologies. Also, the participants’ age range was extensive, with the subjective experience of food and eating varying widely between the young and the elderly.

In the future, it would be interesting to explore further the experiences of younger people, who give particular importance to social interaction and gatherings in food-related leisure contexts, such as fast-food restaurants. Likewise, a gender-based approach could help explore differences in male and female experiences and meanings. Finally, it would also be interesting to explore how food aid equipment (cutlery with special handles, etc.) can mediate subjective experiences.

## 5. Conclusions

This study explored how the eating process is experienced from the viewpoint of people with disabilities, as opposed to current occupational models that prioritise purely nutritional aspects. Our results underline the importance of cultural, social, and contextual factors for an individual’s wellbeing and perceived quality of life. As a result, we stress the importance of re-assessing and re-focusing intervention models, taking into account each person’s context, in particular regarding food and feeding processes. Assessment, intervention, and monitoring models in occupational therapy too often consider the difficulties experienced by patients as inevitable losses, secondary effects, or “sequels” about which nothing can be done. Instead, we suggest that alternative practices need to be developed, based on the appraisal and valorisation of individual experiences of food.

## Figures and Tables

**Table 1:** Participants’ data and types of disability.

	Count (*n*)
**Gender**	
Feminine	6
Masculine	21
**Age groups**	
Under 50	7
50 and over	20
**Disability**	
Brain injury	6
Spinal cord injury	5
Cancer	10
Amyotrophic lateral sclerosis	1
Multiple sclerosis	2
Muscular dystrophy	2
Niemann–Pick	1

**Table 2:** In-depth interview script.

Biography
Clinical history, socio-demographic and socio-economic data
**Illness progression**
Memories prior to impairment, how it started, habits, routines
**The patient’s role**
Accepting the illness; phenomenology; emotions; disruption
**Food and feeding**
Feelings and emotions
Food and social activities
The value of food
Self-sufficiency and changes
**Expectations about the future**
Fears, desires, carers’ expectations
**Treatment itinerary**
Sorrow, narratives, knowledge, chronicity, healing
**Community and personal relationships**
Successes, failures, crises, discrimination

**Table 3:** Categories and subcategories of analysis.

Categories	Subcategories
Feeding	Prior to illness; aid equipment; meals after the illness; food restrictions; food and eating pleasure; eating difficulties
Self-sufficiency	Loss; fear of death; freedom vs dependency; disillusion
Changes	Loss of vital roles; identity; family; environment
Eating (social activity)	Preferences (eating alone); access difficulties; forsaking the practice of eating with others; culinary isolation/loneliness
Discrimination	Shame; loss of capacity; other people’s reactions; stigmatisation; liability
Expectations	Current role; fear of being a burden; carers’ expectations; desires
Habits	Feelings; routines; changes in habits and routines
Meal organisation	Food shopping; food preparation; menu organisation; obligations
Illness progression	Initial stages; understanding; diagnostics; experiences; hospitals; feelings; illness as a “journey”; loss; metaphors; diminished corporality
Social relationships	Exclusion; solitude; jealousy; escape; giving up
